# Comparing classification techniques for identification of grasped objects

**DOI:** 10.1186/s12938-019-0639-0

**Published:** 2019-03-07

**Authors:** Daniel Nogueira, Paulo Abreu, Maria Teresa Restivo

**Affiliations:** LAETA–INEGI, Faculty of Engineering, University of Porto, Porto, Portugal

**Keywords:** Classification, Machine learning, Instrumented glove, Objects identification

## Abstract

**Background:**

This work presents a comparison and selection of different machine learning classification techniques applied in the identification of objects using data collected by an instrumented glove during a grasp process. The selected classifiers techniques can be applied to e-rehabilitation and e-training exercises for different pathologies, as in aphasic patients.

**Methods:**

The adopted method uses the data from a commercial instrumented glove. An experiment was carried out, where three subjects using an instrumented glove had to grasp eight objects of common use. The collected data were submitted to nineteen different classification techniques (available on the scikit-learn library of Python) used in two classifier structures, with the objective of identifying the grasped object. The data were organized into two dataset scenarios: one with data from the three users and another with individual data.

**Results:**

As a result of this work, three classification techniques presented similar accuracies for the classification of objects. Also, it was identified that when training the models with individual dataset the accuracy improves from 96 to 99%.

**Conclusions:**

Classification techniques were used in two classifier structures, one based on a single model and the other on a cascade model. For both classifier structure and scenarios, three of the classification techniques were selected due to the high reached accuracies. The highest results were obtained using the classifier structure that employed the cascade models and the scenario of individual dataset.

## Background

Virtual environments (VE) have been received significant attention when prepared for diagnoses and treatments, for example, of motor and speech e-rehabilitation in patients [1].

Systems intended for e-rehabilitation through the use of VE can be used to monitor and store patient performance data. These data can be used by professionals to assess the progress of patients and to compare them with conventional therapies. Besides, a large number of scenarios and activities can be implemented with different objectives applied in the treatment of the most different difficulties in the area of healthcare [2].

For instance, Horváth et al. [3] propose a VE application for the treatment of patients. In this work, a VE called Virtual Everyday Life Activities (ELA) is presented as a tool for the use in the treatment of patients with cognitive, speech and neuropsychological disorders. Aphasia is an example.

Aphasia is defined as a communication difficulty caused by a focal or degenerative lesion in the areas of the brain responsible for the language, creating problems of expression, comprehension, reading, and writing [4]. Aphasia is caused, for instance, by stroke, head trauma, tumors of the central nervous system, intoxications or infectious and neurodegenerative diseases [5].

The severity of this disorder is related to the extent of the affected area of the brain. The patient may improve rapidly if the damage has not been extensive. However, if there is significant brain damage, the problem can result in severe and lasting disability.

Different patterns of aphasia are related to the location of the brain injury: global aphasia [6], Broca’s aphasia [7], mixed non-fluent aphasia [8], Wernicke’s aphasia [9], primary progressive aphasia [10], and anomic aphasia [11], the patient has difficulty finding words, mainly nouns, and verbs, making continuous discourse difficult. It reaches both spoken and written communication.

Others studies based on the use of technology applied to the treatment of aphasia are shown in Table 1.

**Table 1:** Examples of studies based on the use of technology applied in aphasia

Authors	Work
University of London and Stroke Association	Development of a multi-user online virtual world for practicing speech and communication [12]
Macoir et al.	Review of technology-based aphasia treatments and highlight the critical determinants for the success of treatments [13]
Marshal et al.	Feasibility study of e-rehabilitation systems used in the treatment of patients with aphasia [14]
Roper et al.	Analysis of the benefits and limitations of using a system based on gesture therapy for people with severe aphasia [15]
Lanyi et al.	Software package for an interactive virtual world to assist the speech therapy and the capacity of orientation for aphasic patients [16]

Inspired by those approaches to the use of technology in e-rehabilitation and e-training and, in particular, for the aphasia problem, the use of an online collaborative rehabilitation environment, an instrumented glove, and also integrating an artificial intelligence algorithm has been considered.

The present work results from studies within this context.

CORe, the Collaborative Online Rehabilitation environment, was developed with the main goal of providing an environment to collect and store data from multiple instrumented systems within the field of e-Health, in particular for rehabilitation and occupational therapy also, allowing multi-user interaction [17].

**Fig. 1:**
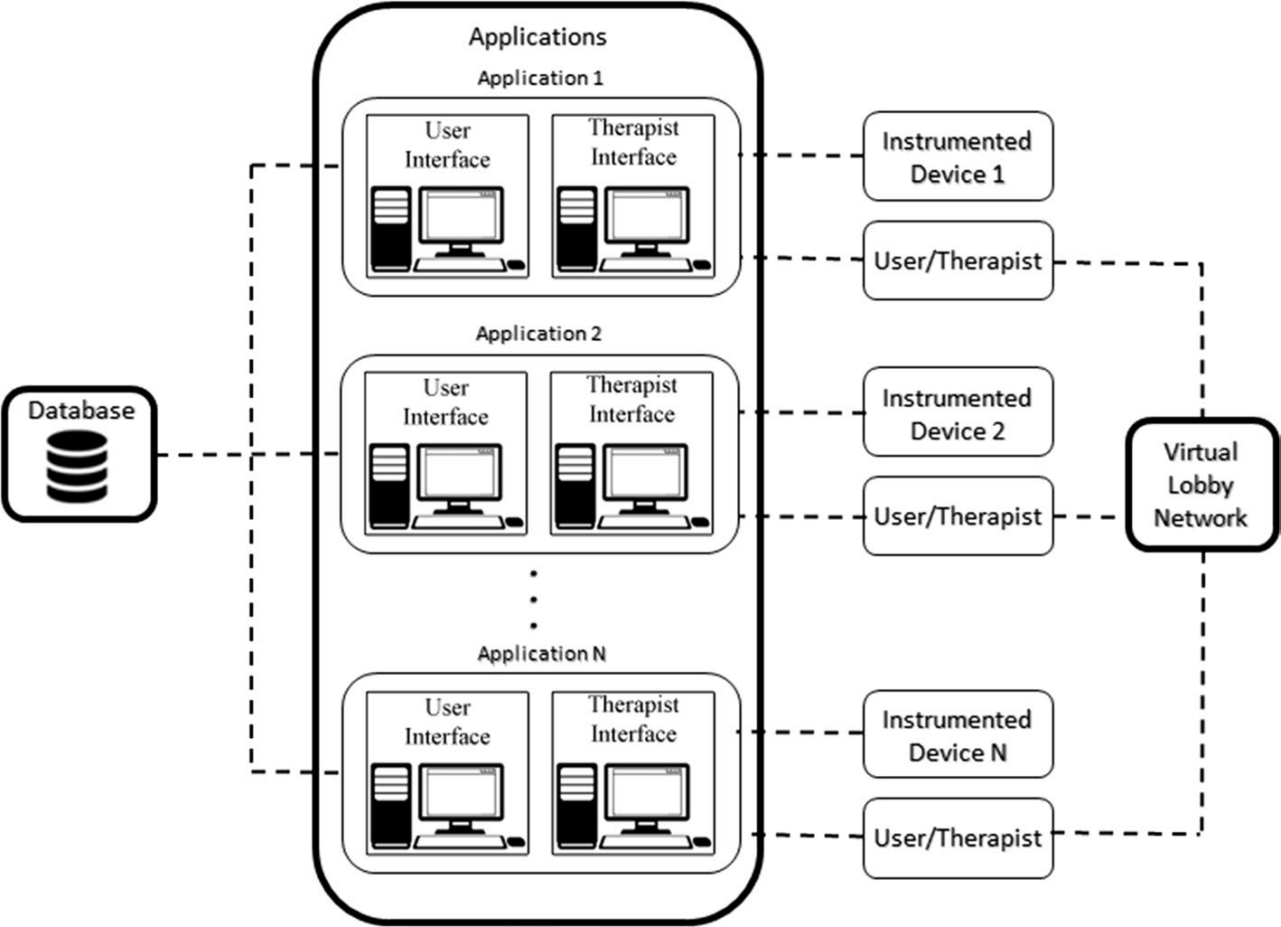
System architecture

CORe has been developed based on a system architecture with three main elements, as shown in Fig. 1:

- Database: a remote database is used to store all the data collected from the different applications;
- Applications: dedicated application for each instrumented device, providing a user interface and a therapist interface;
- Virtual Network Lobby: management of the online collaborative multi-user interaction.

An application of an instrumented glove based on the use of CORe as VE, for the treatment of aphasia, was developed using a commercial instrumented 5DT Glove from 5DT Technologies. The application aims to identify a set of objects commonly used for a person through grasping.

In this paper, machine learning (ML) techniques are tested with the objective of verifying the performance of different classifiers applied to the identification of grasped objects based on data generated by 5DT instrumented glove and collected through CORe. Therefore, this work becomes relevant because it can be a building block for e-rehabilitation and e-training exercises used for aphasia recovering.

A set of supervised learning multiclass classifiers from scikit-learn, a ML library for Python, was tested. Each classifier was analyzed using eight different objects. Three users (one male under 30 years old, one male between 30 and 50 years old and one female over 50 years old) grasped a set of objects using the instrumented glove, following a defined procedure, generating data for training and testing purposes. This study was carried out in an engineering lab environment.

This paper is organized as follows: “CORe implementation” section describes the VE used to integrate the instrumented glove for data acquisition and storage. The set of objects used and the procedures to collect data are shown in “Data collection” section. “Classification techniques” section explores the ML techniques tested in this work. “Methods, results and discussion” describes the methodology used in this work, the results obtained using the metrics adopted and discussions about the structures and scenarios used. “Conclusions” section presents the conclusion.

## CORe implementation

The CORe implementation, using the Unity engine, presents a set of features including:

- Integration of different health monitoring devices: allows patients to use devices in order to carry out e-rehabilitation exercises represented on a virtual environment, promoting e-rehabilitation exercises at home;
- Local and remote storage of data collected by health monitoring devices during activities for later analysis and reproduction;
- Real-time and remote view of e-rehabilitation activities: allows a single therapist to connect with many users in a virtual lobby;
- Gamification in e-rehabilitation: offers game-like activities that take advantage of engagement and motivation for matching the task demands with appropriate feedback and interactive elements;
- Multiplatform: the software environment supports Windows, Android and also WebGL making the stored data remotely accessible anytime, everywhere and for everyone (CORe has not been developed to run on Unix OS).

The software implementation that follows the three main elements of the system architecture are further described.

### Database

**Fig. 2:**
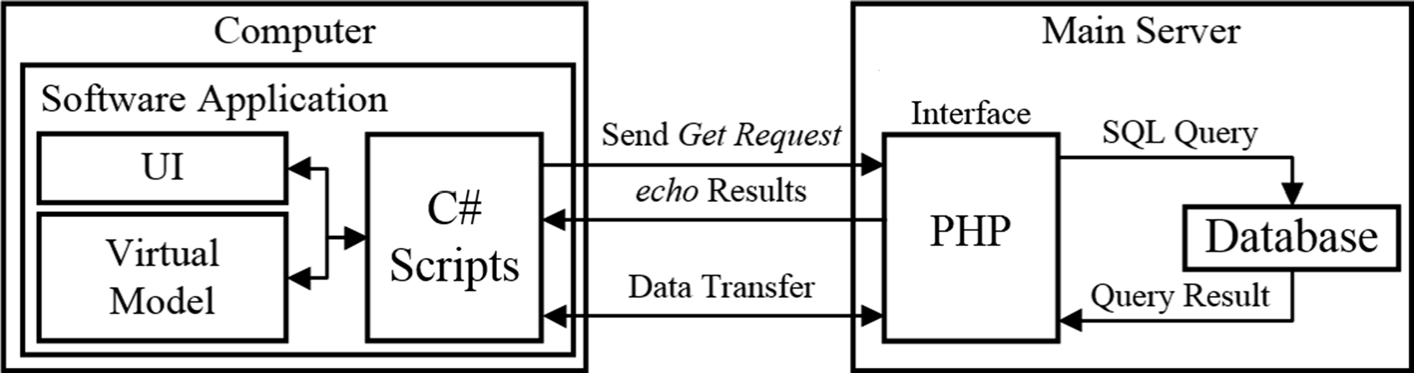
Diagram of the communication between the e-rehabilitation software application and the database

The database is running on a main server for storing the data from the three users and their e-rehabilitation activities. This database runs an open source MariaDB [18] (database servers) implementation. Hypertext Preprocessor (PHP) was used as an interface in order to access the database data, as shown in Fig. 2.

Queries to the database are triggered from a WWWForm (helper class to generate form data to post to web servers) element in Unity’s C# script that sends the variables and values of the query through a GET method into the PHP script. The PHP is called by the C# script and connects to the database, executes the Structured Query Language (SQL) and fetches its result which is encoded in Javascript Object Notation (JSON). Therefore, it sends the JSON encoded string back to the C# script where a parse JSON reconstructs the data. Uploading datafiles into the server or streaming data for replaying a e-rehabilitation activity directly from the server also use a PHP script as an interface.

When there is an inability to connect into the remote database, the data can be stored on a local database. This local database is implemented on each machine running any of the different applications.

### Applications

The software applications were built using the Unity engine, allowing support for different platforms, such as Windows, Android or iOS, enabling the development of a platform independent software. Different applications were developed for each instrumented device. These developments took into consideration the feedback given from specialists in the different fields of health and therapy to ensure the effectiveness of their use. Each application provides a set of activities, an user and a therapist graphical interface, the communication protocols for the specific instrumented device and the connection with the database and the virtual network lobby.

Software libraries were developed that use common communication protocols and were integrated into the software application as pre-compiled libraries. The following communication protocols were implemented:

- Universal Serial Bus (USB) communication: assured by a C++ precompiled dynamic-link library (DLL) were the commands for transferring data can be specified for each instrumented device;
- Bluetooth Low Energy (BLE) characteristic: Java precompiled Android Archive Resource (AAR) implements services to scan for BLE devices and to establish a connection and read data from predefined Generic Attributes (GATT) characteristics;
- Microchip Wireless protocol MiWi (via USB dongle): an alternative for wireless communication protocol designed by Microchip Technology based on the IEEE 802.15.4 standard. It is designed for low data transmission rates and short distances, offering lower latency and higher bandwidth when compared with BLE. This method requires a USB dongle in order to receive the MiWi encrypted packages from the instrumented devices.

Currently, the developed applications support the use of instrumented gloves, handle devices and inertial devices for e-rehabilitation and e-training.

## Data collection

The aphasia problem can be helped with the development of an application for the identification of grasped objects to be used either as an evaluation or e-training tool. For this, it was envisage a procedure where the patient has to grasp common objects using an instrumented glove, and the system automatically identifies the object using a given classification technique.

The conducted study involves data collected from an instrumented glove and the use of classification techniques for the identification of object in this grasping task, based on Heumer’s classification method [19]. CORe was used as VE to collect the data. The 5DT Glove provides data from its five sensors.

**Fig. 3:**
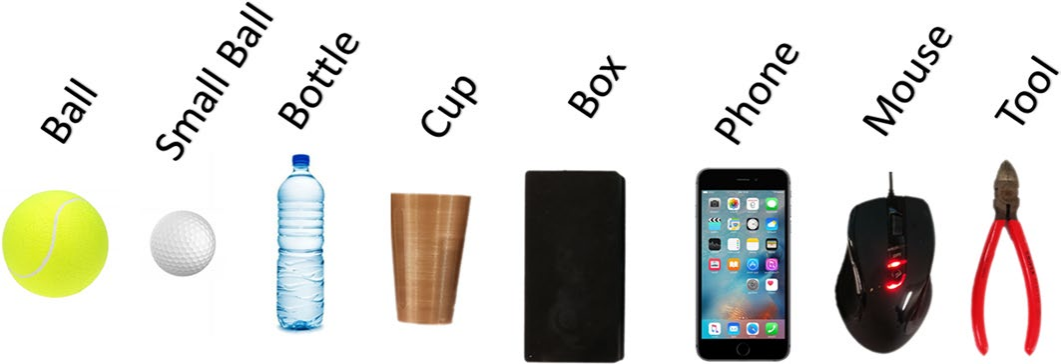
Set of objects

A set of eight objects used is shown in Fig. 3.

The set of objects were split into four groups according to their shape as shown in Table 2. These objects were chosen so that different weight, size, and shape were considered and, with this, different forms of grasping.

**Table 2:** Division of shape group

Shape groups	Objects
Spherical	Ball and small ball
Cylindrical	Bottle and cup
Rectangular	Box and phone
Others	Mouse and tool

The procedure to collect data involves grasping and lifting the objects from the table having the arm in a neutral position. Three users were involved. The grasp period was set to 5 s for data collection. The object was then released and placed back on the desk. This procedure was repeated 100 times for each object, resulting in a total of 800 sets of sensor inputs for each user (totalizing, for three users, 2400 sets of sensors inputs). The data were splited into two sets, one for training (70%) and another for test (30%).

## Classification techniques

ML techniques were used to identify objects, manipulated by an instrumented glove. Within the data analytics field, ML is an area with a growing recognition that can play a critical role in a wide range of applications such as data mining, natural language processing, and image recognition, offering potential solutions in all these domains [20].

In the rehabilitation field, ML techniques can be used to create classification models with data collected from different instrumented devices to identify features and parameters to be used on the evaluation of patient condition [21]. The classification models are created by classifiers that can be divided into three types: supervised, semi-supervised and unsupervised.

The supervised classifier uses a set of labeled data that has its known output. Thus, the classification is based on the existence of a relation between the input and output. The unsupervised classifier allows deriving a structure of data for which the effect of variables is not necessarily known. Thus, during training, the outputs for each entry are not known. Semi-supervised classifiers are a mixture of supervised and unsupervised classifiers. Therefore, output labels are known, but not in entirety.

Python [22] was used as software to implement multiclass classifiers, and scikit-learn [23] was used as a library. The scikit-learn library provides ML functions that are easily implemented. These functions perform complex tasks that are inherent in ML techniques, for instance: cost function calculation, gradient descent, confusion matrix, etc. The scikit-learn, also, was used to dataset splitting into training and test sets.

In this work, all the supervised and semi-supervised classifiers from scikit-learn were used: Bagging [24], Decision Tree (Gini Index and Entropy Function as metric) [25], k-Nearest Neighbors (kNN) [26], Linear [27] and Quadratic Discriminant Analysis [28], SVM (Linear Support Vector Classification—SVC) [29], Logistic Regression (with and without Cross Validation—CV) [30], Multi-Layer Perceptron (MLP) [31], Naive Bayes (Bernoulli and Gaussian metrics) [32], Nearest Centroid [33], Radius Neighbors [34], Random Forest [35], Ridge (with and without CV) [36], Label Propagation [37], and Label Spreading [38]. Despite Label Propagation and Label Spreading are semi-supervised classifiers, they were used as supervised classification techniques according to the characteristic of the dataset.

## Methods, results and discussion

The multiclass classification techniques were tested in two scenarios and with two classifier structures, using three models. The first scenario (universal use) uses data from all users (three users) for training and testing. The second scenario (personalised use) uses data collected from each user to define a personalised training model, to be used with each user.

The training and test data were chosen randomly. The choice of training and test data and classification process occurred 100 times. The reported values (Tables 3 and 4) of training and test time and accuracy are average values. These values considered using the first scenario (universal use).

**Table 3:** Training and test time of model M0, and accuracy of the first classifier structure

Classification technique	First classifier structure scenario 1 (universal use)		
	Training time (ms)	Test time (ms)	Accuracy (%)
Bagging	411.62	15.68	91.9
Decision tree (entropy)	7.2	0.40	55.7
Decision tree (Gini)	5.8	0.30	86.6
kNN	1.36	4.75	91.5
Linear discriminant analysis	10.43	0.24	66.8
SVM (linear SVC)	144.81	0.53	66.1
Logistic regression	41.49	0.42	67.8
Logistic regression CV	915.77	0.48	67.8
MLP	3113.93	0.72	83.5
Naive Bayes (Bernoulli)	1.57	0.35	52.2
Naive Bayes (Gaussian)	1.62	0.86	74.0
NearestCentroid	1.19	0.49	67.9
Quadratic discriminant analysis	3.09	0.76	87.0
Radius neighbors	1.17	146.89	47.5
Random forest	*208.87*	*14.38*	*93.2*
Ridge	2.9	0.22	64.2
Ridge CV	3.05	0.33	64.4
Label propagation	*56.31*	*16.38*	*93.2*
Label spreading	*99.53*	*16.90*	*93.2*

Classification technique with better results are in italic

**Table 4:** Training time of models M1 and M2 and test time and accuracy of the second classifier structure

Classification technique	Training time (ms)		Second classifier structure scenario 1 (universal use)	
	Model M1	Model M2	Test time (ms)	Accuracy
Bagging	392.18	276.36	39.74	95.9
Decision tree (entropy)	5.16	6.82	10.96	82.8
Decision tree (Gini)	5.04	5.14	10.49	92.0
kNN	0.84	0.78	19.72	94.7
Linear discriminant analysis	5.62	6.96	11.35	67.1
SVM (linear SVC)	146.66	202.21	12.47	69.1
Logistic regression	16.21	59.15	11.58	73.6
Logistic regression CV	314.46	1320.23	11.81	74.0
MLP	2478.57	2886.71	12.07	90.5
Naive Bayes (Bernoulli)	1.46	1.04	11.73	56.0
Naive Bayes (Gaussian)	1.5	1.04	12.09	85.3
NearestCentroid	1.02	0.6	11.49	64.3
Quadratic discriminant analysis	1.52	1.4	11.87	82.5
Radius neighbors	1.14	0.82	247.42	54.7
Random forest	*199.41*	*158.87*	*36.58*	*96.6*
Ridge	1.79	1.28	11.76	60.9
Ridge CV	2.64	2.38	11.42	61.0
Label propagation	*56.54*	*57.31*	*46.22*	*96.7*
Label spreading	*97.28*	*99.82*	*45.64*	*96.3*

Classification technique with better results are in italic

**Fig. 4:**
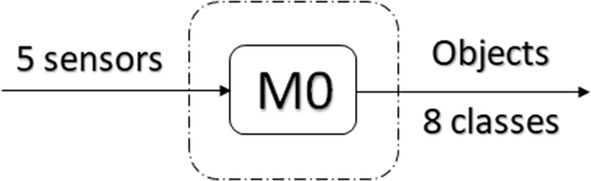
First structure for object classification

The first classifier structure (Fig. 4) uses the model M0. This model uses the data from the five sensors glove as features to classify the objects.

The results using the first classifier structure (based on model M0) are presented in Table 3.

**Fig. 5:**
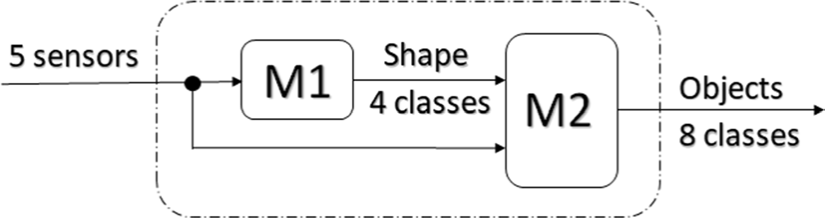
Second classifier structure of objects classification using M1 and M2 models

Three of the selected classifiers revealed better performance compared with the others, regarding accuracy. However, for any of these three, the accuracy value was under 95%. In order to get better classification results, it was decided to introduce an additional feature based on the object shape (as suggested by [19]). This suggested introducing a new classifier structure using two models M1 and M2 (Fig. 5).

The model M1 uses the same features of model M0 to classify the objects’ shape into four classes (spherical, cylindrical, rectangular, and others). Model M2 uses six features (the shape feature from the model M1 output and the data from five sensors of the glove) to classify the object.

Table 4 shows training time for models M1 and M2 and the test time and accuracy of second classifier structure.

As shown in Tables 3 and 4, the classifiers structures, Random Forest, Label Propagation and Label Spreading were the best classification techniques in terms of accuracy. However, the second classifier structure had higher accuracy than the first classifier structure.

Comparing the performance of the two classifier structures (M0 and M1 + M2), the average of Random Forest training time was 3.3 times higher than Label Propagation and 1.9 times than Label Spreading. However, the test time using Random Forest was shorter than using Label Propagation (14% in model M0 and 26% in the M1 + M2) and using Label Spreading (17% in model M0 and 25% in the M1 + M2).

The three best classification techniques obtained using the first scenario (universal use) were chosen to be tested and compared with the second scenario (personalised use). In this scenario, data from each user were, also, randomly divided into training and test datasets. Table 5 compares the test time and accuracies of both scenarios using the two classifier structures.

**Table 5:** Testing times and accuracies—both scenarios

Classification technique	First classifier structure (M0)				Second classifier structure (M1 + M2)			
	Scenario 1 (universal use)		Scenario 2 (personalised use)		Scenario 1 (universal use)		Scenario 2 (personalised use)	
	Test time (ms)	Acc. (%)	Test time (ms)	Acc. (%)	Test time (ms)	Acc. (%)	Test time (ms)	Acc. (%)
Label propagation	16.38	93.2	2.14	94.2	46.22	96.7	4.13	99.0
Label spreading	16.90	93.2	2.28	94.0	45.64	96.3	4.33	98.0
Random forest	14.38	93.2	8.26	95.0	36.58	96.6	4.45	99.0

The accuracy obtained with the second scenario (personalised use) was better than with the first scenario (universal use) independently of the considered classifier structure. On average, the accuracies increased 1.2% for the first classifier structure (M0) and 2.1% for the second classifier structure (M1 + M2).

Looking at the proposed classifier structured (M0 and M1 + M2) the second one allows to achieve better accuracies, independently of the considered scenarios. This is due to the more complex structure that uses more features implying a slight increase in test time.

The test time was shorter for the second scenario (personalised use), in both classifier structures. Although the test time under Scenario 2 within second classifier structure for Label Propagation (4.13 ms) and Label Spreading (4.33 ms) were almost twice to the respective ones within the first classifier structure (2.14/2.28 ms), the time values are in the range of 5 ms.

### Confusion matrices

**Fig. 6:**
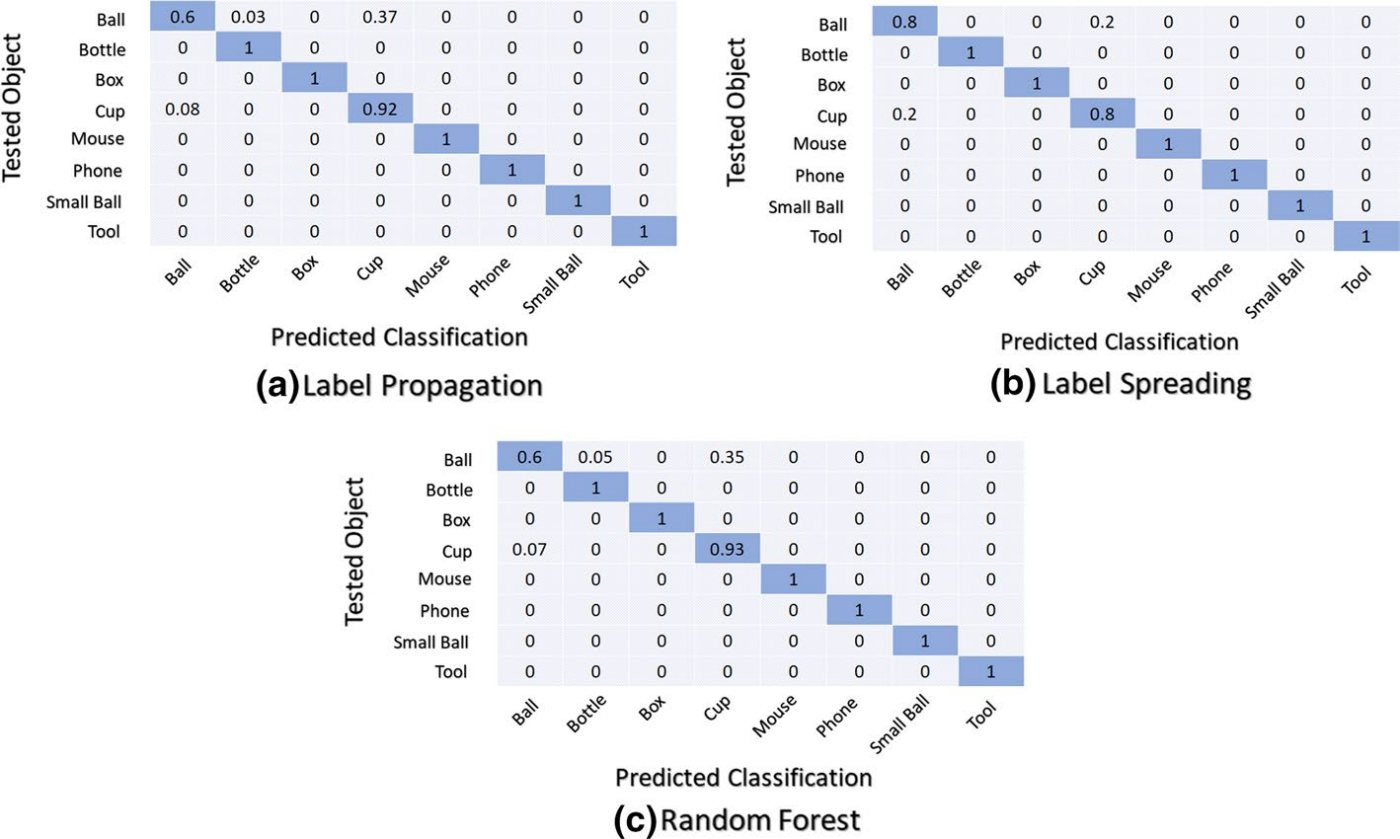
Normalized confusion matrices: first classifier structure (M0) using the first scenario (universal use)

The confusion matrices of the three best classification techniques in both classifier structures used within both scenarios are presented and analyzed. The confusion matrix is used to identify the behaviour of a classifier on a given set of data for which the true values are known. It shows for each tested object the results of the predicted class. Figure 6 presents the average normalized overall confusion matrices for the first classifier structure (M0) using the first scenario (universal use).

**Fig. 7:**
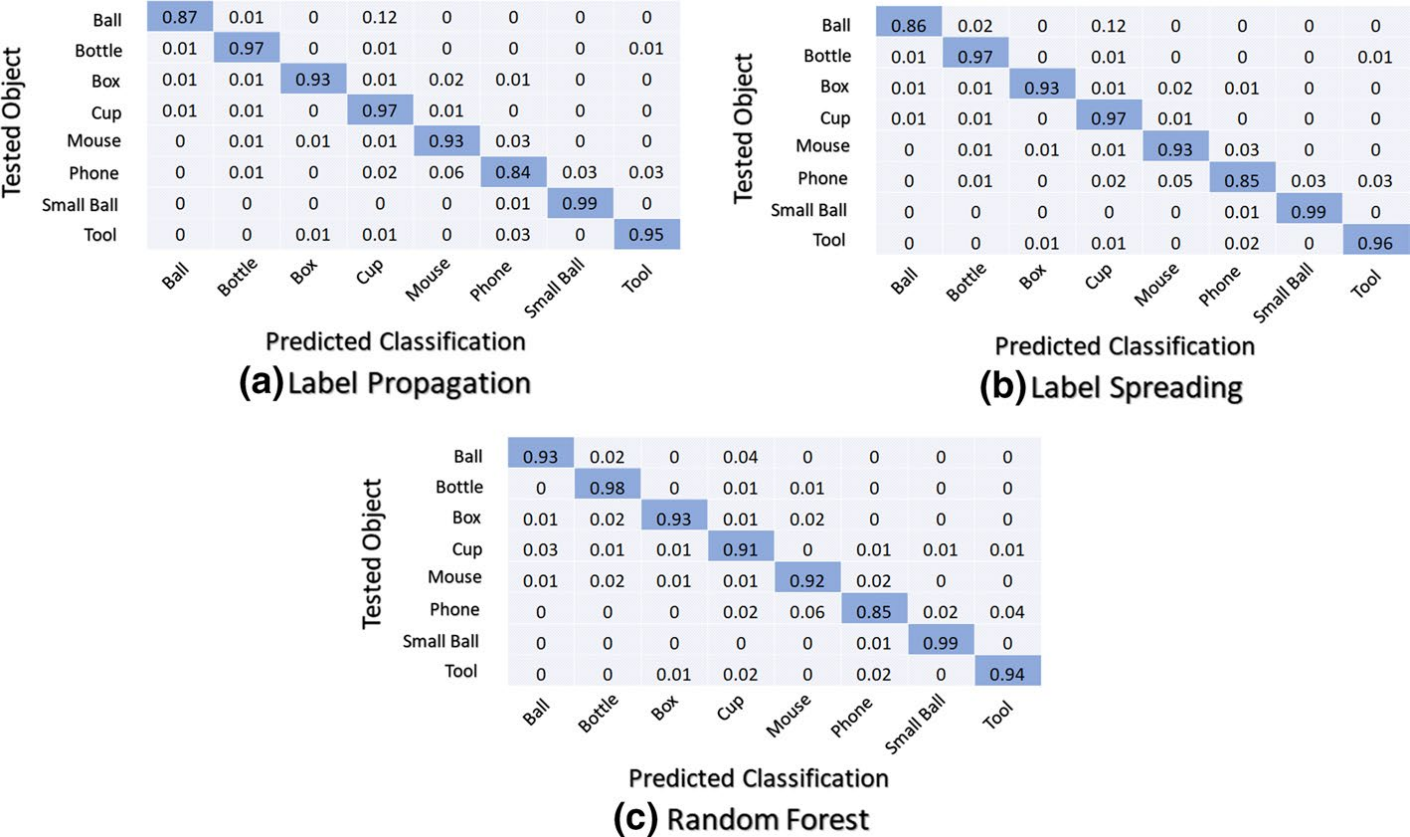
Normalized confusion matrices: first classifier structure (M0) using the second scenario (personalised use)

The average normalized overall confusion matrices for the first classifier structure (M0) using the second scenario (personalised use) is shown in Fig. 7.

**Fig. 8:**
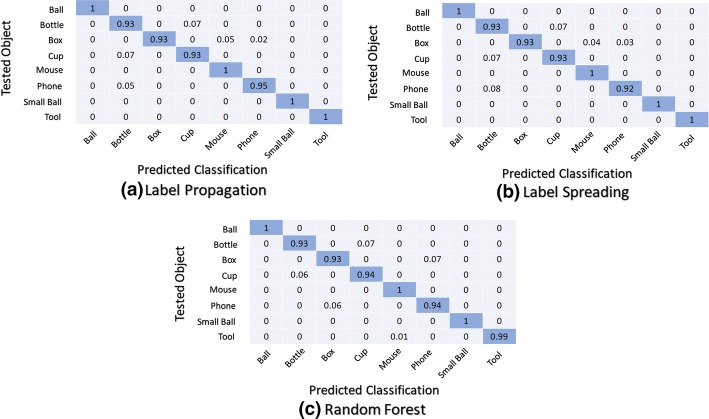
Normalized confusion matrices: second classifier structure (M1 + M2) using the first scenario (universal use)

The average normalized overall confusion matrices for the second classifier structure (M1 + M2) using the first scenario (universal use) is shown in Fig. 8.

**Fig. 9:**
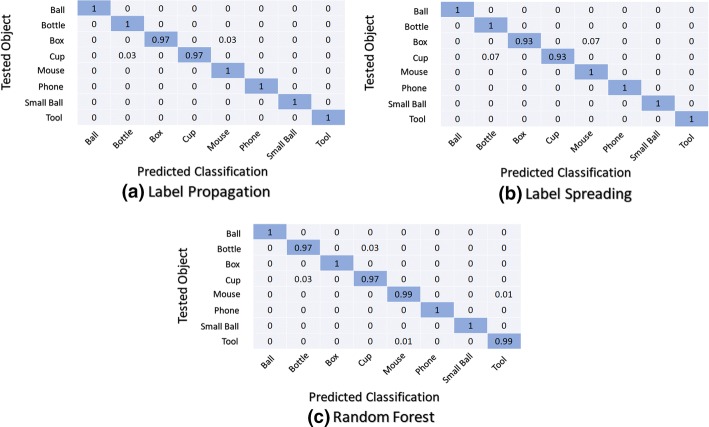
Normalized confusion matrices: second classifier structure (M1 + M2) using the second scenario (personalised use)

Figure 9 shows the average normalized overall confusion matrices for the second classifier structure (M1 + M2) using the second scenario (personalised use).

Observing all the confusion matrices for the second classifier structure (M1 + M2), large errors occur within the classification of objects of the same shape when compared with the first classifier structure (M0). However, in the case of M0, the confusion matrices spread errors among a higher number of different objects.

The confusion matrices show that considering the data from the second scenario (personalised use) the classification errors occur with a small number of objects independently of the considered classifier structure. The second classifier structure (M1 + M2) presents better accuracy, with null classification errors for some objects, independently of classification techniques. Also, the second scenario (personalised use) presents the best results.

## Conclusion

This paper explores and compares different classification techniques for identification of grasped objects. The implementation of ML techniques (from the scikit-learn library) envisages the use of an application to carry out e-rehabilitation and e-training exercises for different pathologies, as in aphasic patients. A commercial instrumented glove (5DT) was used within a developed VE (CORe) that supports data acquisition and storage.

For the data analyzed and the tested classification techniques, the following conclusions were shown:

- the Label Propagation, Label Spreading, and Random Forest classification techniques present the best accuracies (99%, 98% and 99%, respectively);
- for the two considered classifier structures (M0 and M1 + M2), the second (M1 + M2) presents the best accuracies (98% to Label Spreading and 99% to Label Propagation and Random Forest);
- for the two analyzed scenarios (universal and personalised use), the use of personalised approach shows higher accuracies. The average accuracy of the three selected classification techniques using the first classifier structure is 94.4%. For the same personalised scenario the average accuracy of the same selected classification techniques using the second c1assifier structure is 98.6%;
- for the second scenario (personalised use), classification errors mainly occurs in objects of the same shape.

As future work, the following aspects were identified:

- To increase the set of objects to be identified (since up to now eight different objects divided into four type of shapes were considered);
- To include the use of other instrumented gloves with higher number of sensors for comparison studies (5DT Glove used has only five sensors);
- To extend the work for clinical trials following our contacts with experts in aphasia area (as the expected follow up for an engineering laboratory prototype development process).
